# A Low-Cost, Rapidly Integrated Debubbler (RID) Module for Microfluidic Cell Culture Applications

**DOI:** 10.3390/mi10060360

**Published:** 2019-05-30

**Authors:** Matthew J. Williams, Nicholas K. Lee, Joseph A. Mylott, Nicole Mazzola, Adeel Ahmed, Vinay V. Abhyankar

**Affiliations:** 1 Department of Biomedical Engineering, Rochester Institute of Technology, Rochester, NY 14623, USA; mjw3257@rit.edu (M.J.W.); nkl8768@rit.edu (N.K.L.); jam9795@rit.edu (J.A.M.); nxm6619@rit.edu (N.M.); 2 Microsystems Engineering, Rochester Institute of Technology, Rochester, NY 14623, USA; aa5925@rit.edu

**Keywords:** microfluidics, bubble removal, fluid-induced wall shear-stress (WSS), mechanobiology

## Abstract

Microfluidic platforms use controlled fluid flows to provide physiologically relevant biochemical and biophysical cues to cultured cells in a well-defined and reproducible manner. Undisturbed flows are critical in these systems, and air bubbles entering microfluidic channels can lead to device delamination or cell damage. To prevent bubble entry into microfluidic channels, we report a low-cost, Rapidly Integrated Debubbler (RID) module that is simple to fabricate, inexpensive, and easily combined with existing experimental systems. We demonstrate successful removal of air bubbles spanning three orders of magnitude with a maximum removal rate (dV/dt)_max_ = 1.5 mL min^−1^, at flow rates required to apply physiological wall shear stress (1–200 dyne cm^−2^) to mammalian cells cultured in microfluidic channels.

## 1. Introduction

In the 1990s microfluidic systems gained popularity in analytical “lab-on-a-chip” platforms owing to unique microscale capabilities including robust sample routing, rapid multiplexed analysis, and laboratory portability [1,2]. Over the past two decades, these benefits have been extended to cell culture applications where favorable scaling effects (e.g., laminar flows, high surface to volume ratios, and short diffusion distances) have been leveraged to create physiologically-relevant microenvironments featuring precisely controlled biochemical and biophysical stimuli [3,4,5,6]. In these microscale systems, undisrupted flow is required to deliver cell culture media, maintain long-term cell viability, and control cellular-scale cues [7,8]. Unwanted bubbles that enter microscale channels can get trapped and reduce media perfusion rates or cause pressure buildups that precipitate device failure [9]. In addition, bubbles flowing over adherent cells in culture have been shown to cause direct damage to cell membranes through exposure to dynamic air-liquid interfaces [10,11,12]. Given the challenges associated with unwanted bubbles entering microfluidic systems, several mitigation strategies have been developed.

Bubble removal can be divided into two general approaches, (i) bubble traps and (ii) debubblers. Traps focus on guiding bubbles to a containment reservoir before they enter the cell culture region; traps leverage the buoyancy of air bubbles and can be vented with an external vacuum source as the reservoir capacity is reached [9,13,14]. Debubblers remove bubbles via differential transport properties of liquid and air through gas-permeable membranes. For example, Xu and coworkers removed sub-microliter bubbles using a microchannel covered with a porous, hydrophobic acrylic membrane; as the flow stream made contact with the membrane, bubbles were vented through the pores in the membrane and produced a bubble-free solution downstream [15]. Similarly, van Lintel et al. developed an in-line debubbler connected directly to flow tubing using a cartridge with an embedded microporous polytetrafluoroethylene (PTFE) membrane and demonstrated removal of bubbles greater than 5 µL [16]. Lui and coworkers reported a membrane-based debubbler that was integrated into an arrayed microfluidic chip and used to prevent microliter sized bubbles from entering the detection zone of a polymeraze chain reaction (PCR) based assay [17]. Current techniques have successfully removed nanoliter to microliter volume bubbles with maximum removal rates (dV/dt)_max_ ranging from 0.5 μL min^−1^ to > 2 mL min^−1^.

Although myriad debubblers have been reported in the literature, they are often complex and highly application-specific; thus, integration into more general microfluidic systems represents a significant implementation barrier. To address hurdles related to both complex fabrication and integration of debubblers, we introduce a simple workflow to create a rapidly integrated debubbler (RID) module that can be easily combined with existing microfluidic systems. Key practical features of the RID module include (i) an accessible fabrication process with rapid assembly (<2 min), (ii) low device cost (<$0.50 at lab prototype level), and (iii) press-to-fit tubing connections to simplify component integration. Controlled shear stimulation of cultured cells is a hallmark capability in microfluidic systems that enables quantitative correlation between applied fluid-induced wall shear stresses (WSS) and cellular responses including endothelial cell alignment [18], calcium signaling [19], and barrier formation [20,21]. Thus, we validated RID performance by characterizing bubble removal capabilities ranging from nanoliter to microliter volume bubbles at flow rates required to apply physiological WSS to cultured mammalian cells within standard geometry microfluidic channels.

## 2. Materials and Methods 

### 2.1. Rapidly Integrated Debubbler (RID) Fabrication and Assembly 

**Figure 1:**
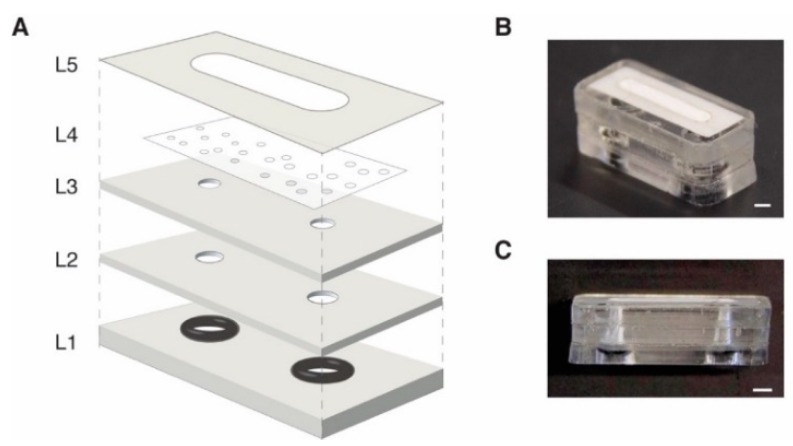
(**A**) Exploded view of the RID module. L1–L3 are laser cut from polymethymethacrylate (PMMA). L1 and L2 have pressure sensitive adhesive on the top face to facilitate layer-to-layer lamination. L3 is the tubing stop layer used to prevent damage to L4, the porous, hydrophobic PTFE membrane. L5 is laser cut from polyester film and has PSA on the bottom surface to seal the edges of the PTFE membrane and prevent leakage. After assembly, an O-ring is inserted into the access ports in L1 to provide simple push-to-connect compression sealing to interface the device with tubing for fluid routing. (**B**) Isometric image of assembled RID module. Scale bar = 2 mm. (**C**) Side view of assembled device. Scale bar = 2 mm.

As shown in Figure 1, structural elements (L1–L3) were designed as vector files in Adobe Illustrator (San Jose, CA, USA) and cut from polymethylmethacrylate (PMMA, 2 mm thickness, McMaster-Carr, Aurora, OH, USA) using a 40 W CO_2_ laser (Full Spectrum Laser, H-Series, Las Vegas, NV, USA) with inlet and outlet ports in L1 designed to house #003 rubber O-rings (outer diameter (OD) = 3/16”, inner diameter (ID) = 1/16”, Durometer 70A, McMaster-Carr). Pressure sensitive adhesive films (PSA, 3M MP467, Maplewood, MN, USA) were rolled onto the top surfaces of L1–L2 prior to laser cutting using a cold roll laminator. A polytetrafluoroethylene (PTFE) membrane (0.01 mm pore diameter, 0.1 mm thickness, Sterilitech Corp, Kent, WA, USA) was cut with a razor blade and placed in contact with L3. L3 (PMMA) was used to protect the PTFE membrane from damage due to improper insertion of the tubing. The 2 mm wide by 12 mm region cut into L5 (polyester film with PSA on bottom surface) defined the degassing region of the device, and the PSA lamination produced liquid-tight sealing between layers. Once all layers were cut in a batch process, individual devices could be assembled in less than 2 min. The packed O-ring assembly allowed simple press-to-fit connection with 1/16” OD tubing, and the addition of barbed end fittings (Cole Parmer, Vernon Hills, IL, USA) allowed attachment to flexible tubing. The overall footprint of the RID module was 10 mm × 18.5 mm (W × L). 

### 2.2. RID Operating Principle

The operating principle of the RID module is based on total surface energy E_total_ minimization as the vapor (v) phase (e.g., bubble) and liquid (l) phase interact with the solid components of the PTFE membrane. The change in total surface energy, ΔE_total_, can be expressed as the change in interfacial area, ΔA, during the attachment of vapor to the membrane, and the difference in surface energy (γ) between the solid-vapor (sv) and solid-liquid (sl) interfaces [22]:

(1) $${\Delta E}_{\mathrm{total}}=\Delta A\;{(\gamma }_{\mathrm{sv}}-\gamma_{\mathrm{sl}}).$$

The Young equation relates the solid-liquid contact angle θ_sl_ to the interfacial energies:

(2) $$\gamma_{\mathrm{lv}}\cos\;\theta_{\mathrm{sl}}=\gamma_{\mathrm{sv}}-\gamma_{\mathrm{sl}}.$$

With a hydrophobic material, (θ_sl_ > 90°) the right-hand side of Equation (2) is negative implying that γ_sv_ < γ_sl_; from Equation (1), there is a corresponding energetically favorable decrease in total surface energy. Conversely, for a hydrophilic material (θ_sl_ < 90°), γ_sv_ > γ_sl_ with unfavorable ΔE_total_ > 0. Thus, the hydrophobic PTFE membrane used in the RID module energetically favors the formation of vapor-solid interfaces when compared with a hydrophilic material.

**Figure 2:**
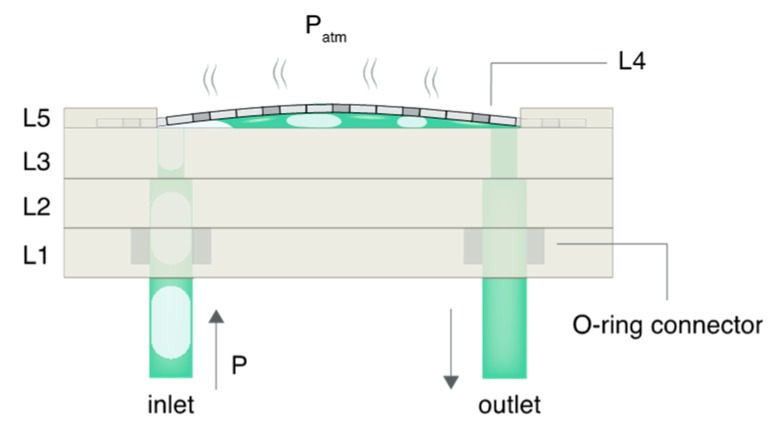
Schematic representation of the RID module in operation. Once the opening pressure P_open_ is exceeded, the PTFE membrane (L4) deflects and exposes a fluidic path connecting the inlet and outlet. Bubbles in the input stream make contact with the hydrophobic PTFE membrane and are removed as a result of the differential pressure between the air-liquid fluid stream and the atmosphere. The liquid is unable to move through the membrane below the critical liquid entry pressure, P_critical_. As air bubbles are removed, liquid plugs recombine to form a single-phase flow exiting the RID module.

As shown in Figure 2, when the pressure from the input fluid stream P > P_open_, L4 deflected away from L3 and exposed a fluidic path from the inlet to the outlet. Bubbles present in the flow stream made contact with L4 and were vented out of the device due to the transmembrane pressure gradient between the segmented flow and the atmospheric pressure, P_atm_. However, liquid could not pass through L4 at pressures less than the critical membrane liquid entry P_critical_, which was determined experimentally. Qualitatively, the P_critical_ is inversely proportional to the effective membrane pore diameter, d, as shown by the Laplace‒Young equation, where θ_max_ is the maximum contact angle between the liquid and membrane pore surface at equilibrium:

(3) $$P_{\mathrm{critical}}{=4\gamma }_{\mathrm{lv}}(\pi -\theta_{\max})/d$$

At P > P_critical_ the pressure from the flow stream exceeded capillary pinning effects and liquid was forced through the pores. At P < P_critical_ liquid segments recombined as bubbles were removed and formed a single-phase flow prior to exiting the RID module. The operating pressure range was defined as P_open_ < P < P_critical_. The upper pressure limit could be increased by using a thicker membrane (due to an increased surface interaction area between the aqueous liquid and hydrophobic PTFE) or by decreasing the pore size (Equation (3)). 

### 2.3. RID Characterization and Automated Image Analysis

To characterize RID bubble removal, individual streams of air and liquid (New Era Pump Systems, Inc Farmingdale, NY, USA) were combined to create segmented flow streams and directed toward the input port of the RID. The test liquid was complete cell culture media Dulbecco’s Modified Eagle Medium /F-12 (DMEM/F12, 10% v/v fetal bovine serum, FBS) with food color added to increase contrast during imaging. Flow streams entering and exiting the RID module were simultaneously recorded (Samsung Galaxy S6) and analyzed with a custom MATLAB image processing script (see supplemental MATLAB code). Black and white binary images corresponding to air and liquid respectively were obtained from the captured video frames. The leading and trailing edges of the segments were identified, and tubing inner diameter along with video frame rate were used to calculate (i) the flow rate Q, (ii) volume of each bubble V_b_, and (iii) V_b_:V_T_ ratio (defined as bubble volume to total volume). A test condition failed when a bubble was visualized in the fluid exiting the device via image analysis. We conservatively report the failure threshold as the lower bound of the 95% confidence interval determined from failure testing of four independent RID devices. The equation WSS = 6 μQw^–1^ h^–2^ (valid for laminar, fully developed flow, and h << w) [23] was used to relate Q to applied wall shear stress (for a bubble-free stream) in a standard geometry microfluidic culture channel with h = 0.1 mm, w = 1 mm, and l = 10 mm (channel volume, V_channel_ = 1 μL). 

### 2.4. RID Opening Pressure P_open_ and Membrane Liquid Entry Pressure P_critical_

A 5 mL fluid reservoir was connected vertically to the input of the debubbler. With the debubbler dry, cell culture media was added to the reservoir to generate a hydrostatic pressure head ΔP = ρgΔh with Δh denoting the difference in height between the media level and the outlet port. Using video analysis, the height difference required to initiate flow through the debubbler was determined and converted to the opening pressure P_open_. Results reported as mean ± standard deviation (SD) from four independent devices.

A syringe pump was used to introduce cell culture medium to the RID, while the outlet port was connected to dead-end tubing. Sensors on either side of the debubbler (Parker Hannifin, Cleveland, OH, USA) measured the pressure across the module as a function of time. Video analysis was used to determine the critical liquid pressure P_critical_ at which fluid was forced through the PTFE membrane. Results reported as mean ± SD from four independent devices. 

### 2.5. Fluid Properties, Contact Angle, and Interfacial Tension Measurements

A glass capillary viscometer was used to measure the viscosity of DMEM/F12 cell culture media with 10% FBS. Fluid density was determined by measuring mass of a known media volume using an analytical balance (Mettler Toledo, Columbus, OH, USA) at 37 °C. A goniometer (Rame-Hart 250, Rame-Hart instrument company, Succasunna, NJ, USA) was used to measure the interfacial tension of cell culture media in air, and the contact angle between the culture medium and PTFE membrane using the hanging drop and sessile drop methods respectively. Results reported as mean ± SD from four independent devices. 

### 2.6. Cell Culture

For routine culture, human umbilical vein endothelial cells (HUVEC) were cultured in DMEM/F12 with 10% v/v FBS using the manufacturer’s protocols. Briefly, HUVECs were enzymatically disassociated using Trypsin/EDTA (Life Technologies, Carlsbad, CA, USA) and sub-cultured when they reached 70% confluence. Cells were centrifuged at 250 g, resuspended in medium, and counted. Cells were plated at 5000–10,000 cells cm^–2^ on Geltrex coated tissue culture flasks. Media was replaced every 24–48 h. 

For bubble exposure studies, a microfluidic channel network was sealed against a glass slide using our previously reported reversibly Sealed, Easy Access, Modular (SEAM) culture platform [24,25]. Glass slides (Fisher Scientific, Hampton, NH, USA) were sterilized in ethanol, rinsed three times in phosphate buffered saline (PBS, Fisher Scientific, Hampton, NH, USA), then allowed to dry in a biosafety cabinet. A PMMA piece with a 15 × 20 mm cavity and embedded magnets (K&J Magnetics, Plumsteadville, PA, USA) was sterilized with ethanol and attached to the glass with PSA. The cavity was treated with a Geltrex solution (Thermo Fisher, 0.1 mg mL^−1^) at 37 °C for 1 h to improve cell adhesion. Excess Geltrex solution was aspirated, and a PDMS microfluidic network was magnetically sealed against the glass substrate. The microfluidic channel network was fabricated using a soft lithography process described previously [24]. HUVECs were seeded at a density of 40,000 cells cm^−2^ and allowed to attach overnight. Cells were then treated with Calcein AM (ThermoFisher, Waltham, MA, USA, 0.5 μM concentration in PBS) and the cell permeable Hoechst 33342 stain (ThermoFisher, 1:2000 dilution in PBS) for 15 min to aid visualization of HUVEC cytoplasm and nuclei respectively. HUVECs in culture were imaged after exposure to a segmented air-liquid stream with flow rate corresponding to WSS of 11.5 dyne cm^−2^ with and without a RID module upstream of the culture device. The RID modules were sterilized with ethanol followed by 3× wash with PBS to remove residual ethanol and allowed to dry before use. 

## 3. Results and Discussion

### 3.1. Characteriation of RID Bubble Removal Capabilities

**Figure 3:**
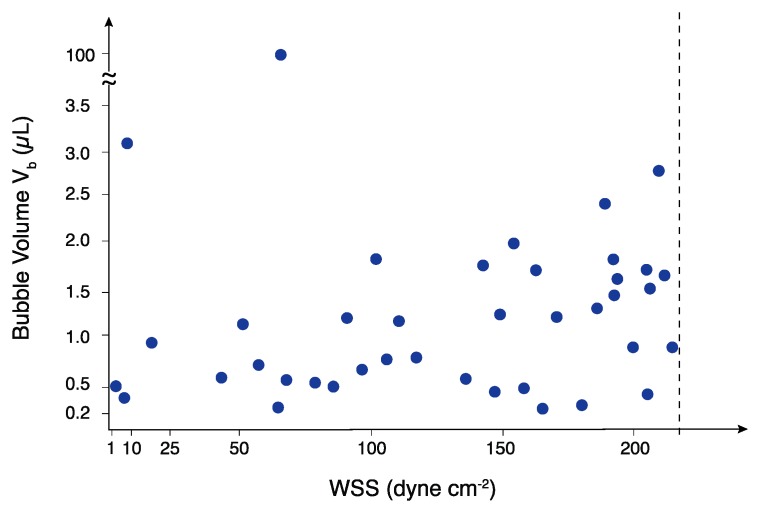
RID module bubble removal results at flow rates corresponding to defined WSS in a microfluidic channel. Each data point represents air bubbles successfully removed from a segmented air-liquid flow with bubble volume V_b_. The dashed line represents the lower bound of the 95% confidence interval for successful device operation (WSS = 213 dyne cm^−2^, Q = 2.6 mL min^−1^). Air bubbles ranging from 200 nL to 100 μL were successful removed and the maximum air removal rate (dV/dt)_max_ = 1.5 mL min^−1^. RID is operational across the physiological WSS range needed for in vitro shear stimulation studies using both human and rodent cells.

To ensure that RID modules could be used for shear stimulation studies relevant to human (1–50 dyne cm^−2^) [26] and rodent cell studies (50–200 dyne cm^−2^) [27], segmented air-liquid streams were introduced at flow rates corresponding to defined WSS in a standard geometry microfluidic channel, h = 0.1 mm, w = 1 mm, and l = 1 cm, with the channel volume V_channel_ = 1 μL. The range of tested bubble volumes was selected to evaluate bubble removal capabilities under common flow disruption scenarios: (i) V_b_ < V_channel_, (ii) V_b_ ≥ V_channel_, and (iii) V_b_ >> V_channel_ (i.e., catastrophic problem with pump or pressure source). A custom image processing algorithm was used to measure V_b_, V_b_:V_T_, and Q. Results from the RID bubble removal testing are summarized in Figure 3. A test condition was considered to be unsuccessful if a single bubble was detected in the outlet tubing via image analysis; therefore, the reported operational range represents conditions where air bubbles were completely removed. 

The RID successfully removed bubbles across three orders of magnitude (200 nL ≤ V_b_ ≤ 100 μL) from segmented flows (0.07 ≤ V_b_:V_T_ ≤ 0.7) including the challenging high Q (high WSS), small V_b_, and high V_b_:V_T_ conditions where dV/dt must be sufficiently high to remove bubbles before they are displaced downstream by the incoming flow. The average WSS at failure = 289 ± 48 dyne cm^−2^ with 95% confidence interval, 213 < WSS < 352 dyne cm^−2^; the dashed line in Figure 3 shows the lower limit of the 95% confidence interval (213 dyne cm^−2^ or Q = 2.6 mL min^−1^), and represents a conservative limit of the operational range. Although the 95% confidence interval is relatively large, the RID reproducibly removed large and small volume bubbles at flow rates corresponding to WSS that span the physiological ranges of both human and rodent cells commonly used to study shear stimulation in vitro. RID (dV/dt)_max_ = 1.5 mL min^−1^, enables rapid removal of bubbles, and is competitive with more complex and specialized debubblers. Traditionally, debubblers are designed for a particular bubble removal operation range; to the best of our knowledge, this is the first demonstration of a rapid debubbler spanning at least three orders of magnitude in removed V_b_ with a single device. It should be noted that V_b_ = 100 μL was the maximum bubble volume tested due to experimental limitations and was not the operational failure limit of the RID. Although bubbles with V_b_ < 200 nL could not be reproducibly generated using our experimental setup, we anticipate that smaller volume bubbles would be removed either at (i) the entrance of the RID module via contact with the PTFE membrane due to the perpendicular orientation of the input tubing or (ii) in the gap between the membrane and PMMA where the bubble interactions with the membrane are energetically favorable. 

**Figure 4:**
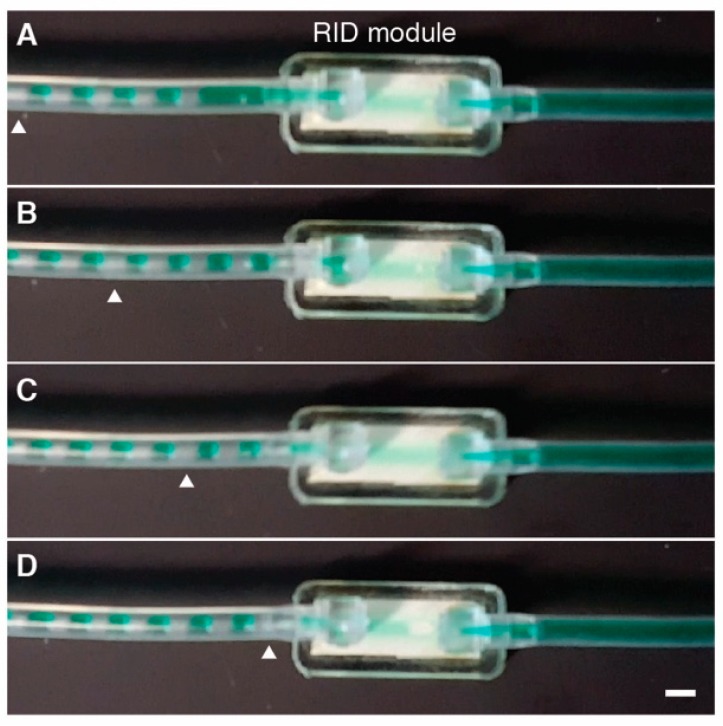
Sequential frames A‒D extracted from video showing bubble removal from an incoming segmented flow stream (bubble volume = 2.2 ± 0.3 μL, Q = 1.1 mL min^−1^, and V_b_:V_T_ = 0.4). Flow is left to right. (**A**) White arrow highlighting bubble at left edge of frame, t = 0 s. (**B**) Segmented flow moving down tube toward RID, t = 0.73 s. (**C**) Bubbles upstream of highlighted bubble enter RID, t = 1.33 s. (**D**) Highlighted bubble close to the entrance of the RID module. Upstream bubbles have been removed and bubble-free flow leaves the module, t = 2 s. Bubble removal rate dV/dt = 440 μL min^−1^. Scale bar = 3 mm.

Figure 4 shows a representative image sequence spanning 2 s and demonstrating removal of air bubbles (V_b_ = 2.2 ± 0.3 μL, V_b_:V_T_ = 0.4) at Q = 1.1 mL min^−1^ (liquid WSS = 90 dyne cm^−2^); flow direction is left to right. Bubbles entering the RID module were removed with dV/dt = 440 μL min^−1^ and single-phase flow exited the device (see supplemental Video S1 “Figure 4 Video”). In addition to uniformly segmented flows, which were used to determine bubble removal in a reproducible way (see supplemental Video S2 “Rapid Bubble Removal”), the RID module was capable of removing polydisperse bubble volumes from incoming flow streams (see Supplemental Video S3 “Polydisperse Bubbles” and S4 “Polydisperse Large Bubbles”.)

### 3.2. Cell Damage Resulting from Bubble Introduction

**Figure 5:**
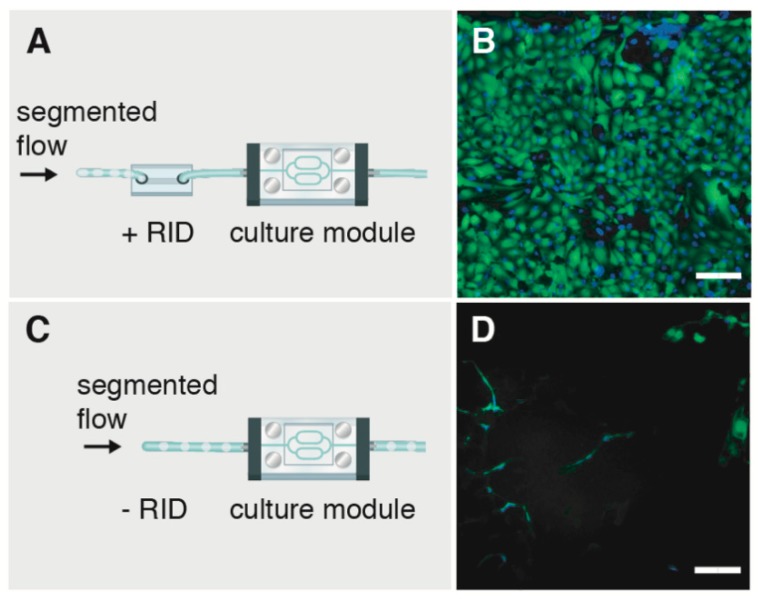
Representative images showing the importance of the RID module to protect against bubble-induced damage. Segmented flows were introduced upstream of the cultured cells (**A**) with a RID module and (**C**) without the RID module in place. (**B**) With the RID in place the cell population remained viable, while without the module (**D**) bubbles entering the channel caused damage to cell membranes. Scale bar = 100 μm.

As shown in Figure 5, we demonstrate the importance of preventing air bubbles from entering a microfluidic channel. To replicate a common flow disturbance where V_b_ ≥ V_channel_, segmented flow streams were introduced upstream of the culture module with and without a RID module in place (see supplemental Figure S1). Flow rate corresponding to physiological WSS of 11.5 dyne cm^−2^ (for bubble-free flow) was maintained for both experimental conditions. Figure 5A,C schematically show the experimental conditions (e.g., segmented flows +RID and −RID), while Figure 5B shows viable cells in the culture channel with the RID module in place. Figure 5D shows that without a RID module, cell membranes appear damaged. The damage can be explained in part by an amplification in applied WSS resulting from a thin lubrication layer present between the bubble and surrounding walls [11,28,29]. WSS amplification is caused by an increased velocity gradient in the thin lubricating layer of thickness b << h as the bubble moves through the channel, compared to the bubble-free condition where WSS = 6 μ Qw^−1^ h^−2^. Under the test conditions, we estimate the amplification factor Φ comparing WSS with and without bubbles (e.g., Φ = WSS_bubble_/WSS) = 55 (see supplemental Equations, table, figure file for details). As seen in Figure 5, the presence of bubbles in a microfluidic channel can dramatically increase WSS from physiological conditions to levels where cell damage can occur. The introduction of the RID module in the flow path prevented bubbles from entering the channel network and supported viable cell culture. RID fabrication workflow enables complete debubbler customization to meet experimental needs and simplify integration into a microfluidic setup. 

### 3.3. RID Operating Pressure Range and Chip-To-World Interconnections

To determine the operating pressure range for the RID (P_open_ < P < P_critical_), we measured (i) the minimum pressure, P_open_, required to deflect the PTFE membrane from the PMMA layer and (ii) the maximum pressure, P_critical_, above which cell culture media was forced through the hydrophobic PTFE membrane pores. The operating pressure range spanned three orders of magnitude with P_open_ = 7.4 ± 0.4 Pa and P_critical_ = 9.7 ± 1.7 kPa. The small opening pressure can be attributed to the limited adhesion between the hydrophobic PTFE and the PMMA surface that must be overcome by the flow stream pressure. P_critical_ is a function of membrane pore diameter; the manufacturer’s reported PTFE pore diameter of 10 μm was sufficient for our applications. However, a membrane with a smaller pore size can be used if a higher P_critical_ is desired (see Equation (3)). It should be noted that the PTFE membranes do not contain well-defined circular pores but are comprised of a fibrous mesh that contain voids between fibers; the relatively large 95% CI for WSS at failure can be attributed in part to inhomogeneities between membranes. 

Microfluidic cell culture platforms often require elements to be connected together to form a fluidic flow path (e.g., a pressure source sequentially connected to a flow damper, cell culture module, and downstream collection vessel). The interconnection problem can be a source of frustration because each component can have different tubing requirements and corresponding mating mechanisms (e.g., barbed, press to fit, or ferrule). A goal of this work was to increase accessibility by providing an integration approach to simplify connections. Here, we implemented a simple connection mechanism via embedded O-rings in the top housing layer of the RID module that form a compression seal against inserted rigid tubing. The press-to-fit O-ring connectors (See Figure 2) were able to withstand pressures of at least 70 kPa (maximum range of pressure sensor); this limit was sufficient to ensure leak-proof operation in our system where the maximum operational pressure P_critical_ was an order of magnitude lower than 70 kPa. The O-ring connector can also be used as an adaptor to insert barbed fittings to connect the RID with flexible tubing, and thus simplify integration into existing microfluidic setups without requiring design modification.

### 3.4. Device Fabrication Workflow

To facilitate customization and improve debubbler accessibility for the microfluidics community, the RID fabrication workflow uses equipment commonly found in a community makerspace or general research laboratory. Our approach incorporates commercially available materials (e.g., PMMA, PSA, and PTFE membranes), and modules can be assembled in less than 2 min when layers (Figure 1) are cut in a batch process. Design prototypes can be easily iterated because the process workflow allows complete control over the device geometry and layer components. With the current design, each module costs less than $0.50 (see Table S1), allowing them to be treated as consumable components. The fabrication process is also amenable to being scaled up using reel-to-reel processing or injection molding techniques once a final design is identified. 

## 4. Conclusions

We have demonstrated a simple fabrication workflow to create a debubbler that can rapidly remove bubble volumes spanning three orders of magnitude from segmented flows at flow rates compatible with those required for microfluidic shear stimulation studies. The fabrication process can be carried out in a general makerspace or research laboratory and the footprint and fluidic interconnections can be customized as needed to fit existing experimental setups. We anticipate that the combination of simple fabrication, integration, and functional capabilities will enable RIDs to be easily implemented into microfluidic applications where the entry of bubbles is undesired.

## Supplementary Materials

Supplemental information: Equations, tables, and figure are available online at https://www.mdpi.com/2072-666X/10/6/360/s1, Video S1: Rapid bubble removal are available online at https://www.mdpi.com/2072-666X/10/6/360/s2, Video S2: Figure 4 Video are available online at https://www.mdpi.com/2072-666X/10/6/360/s3, Video S3: Polydisperse Bubbles are available online at https://www.mdpi.com/2072-666X/10/6/360/s4, Video S4: Polydisperse Large Bubbles, and MATLAB code with user guide are available online at https://www.mdpi.com/2072-666X/10/6/360/s5.
