# Supplementary material for: A Low-Cost, Rapidly Integrated Debubbler (RID) Module for Microfluidic Cell Culture Applications
Source: Micromachines (Basel). 2019 May 30;10(6):360. doi: 10.3390/mi10060360 (PMC6632054; doi:10.3390/mi10060360)
Supplement: Supplementary file 1 [file micromachines-10-00360-s001.zip › Supplemental equations table figure.pdf]

A low-cost, rapidly integrated debubbler (RID) module for microfluidic cell culture applications

Matthew J. Williams, Nicholas K. Lee, Joseph A. Mylott, Nicole Mazzola, Adeel Ahmed, and Vinay V. Abhyankar*

*corresponding author: vvabme@rit.edu

Supplemental Equations, Tables, and Figure

Amplification of fluid-induced wall shear stress with bubble introduction

Applied WSS in a rectangular channel the absence of bubbles can be determined using the relation WSS = $6\mu Q w^{-1} h^{-2}$ for laminar, fully developed flow. In this experiment, Q corresponded to 11.5 dyne cm^{-2} in a microfluidic channel with h = 0.1 mm, w = 1 mm, l = 10 mm. As a first order estimate of WSS amplification in the presence of well-studied Taylor bubbles, (e.g. bubble length > channel width) it is assume a lubrication film is present between the bubble and the surrounding walls [1,2]. In the classical analysis, film thickness is considered to be constant between the wall and the Laplace pressure jump across the curved air-liquid interface at the front of the bubble is neglected. The capillary number Ca = $\mu U \gamma^{-1}$ with bubble velocity U, and interfacial tension γ and relates viscous to interfacial phenomena; for Ca < $5x10^{-3}$, the Bretherton equation can be used to estimate the thickness of the lubrication film: b = $0.643(3\ Ca^{2/3})R_H$ where R_H is the hydraulic radius. For channels with w >> h, R_H simplifies to 0.5h. In our system, μ =$8.24x10^{-4}$ Pa sec, γ = 0.063 N m^{-1}, (both measured at 37°C) and bubble velocity U measured for input flow rate of Q = 140 µL min^{-1}, Ca = $3.06x10^{-4}$ and film thickness b = 0.3 µm. With bubble velocity U, WSS_{bubble} = $\mu U b^{-1}$ and amplification factor Φ = WSS_{bubble}/WSS. In our system, Φ = 55 and is consistent with similar analysis by Lochovsky and coworkers [3].

In-line RID module to SEAM module setup

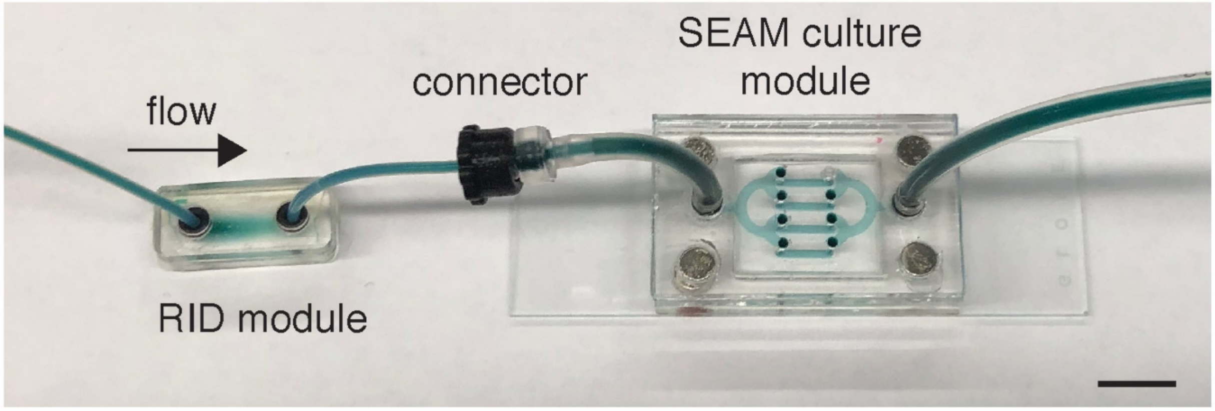

Figure S1. Integration of RID module with SEAM cell culture module using O-ring and barbed ended tubing connectors. Scale bar = 7.5mm.

Table S1 – List of Materials and Estimated Cost Per Device

Material	Cost/mm² [USD]	Total cost per device	Layer designation
PMMA	0.0000569	$0.0316	L1-L3
PSA	0.0000503	$0.0279	L1-L3
PTFE	0.002334	$0.2987	L4
O-rings	0.034 (per O-ring)	$0.0680	L1
transparency	0.0000325	$0.0060	L5
		**$0.43**	

References

1. Bretherton, F.P. The motion of long bubbles in tubes. J. Fluid Mech. 1961, 10, 166.
2. Klaseboer, E.; Gupta, R.; Manica, R. An extended Bretherton model for long Taylor bubbles at moderate capillary numbers. Phys. Fluids 2014, 26, 32107.
3. Lochovsky, C.; Yasotharan, S.; Günther, A. Bubbles no more: In-plane trapping and removal of bubbles in microfluidic devices. Lab Chip 2012, 12, 595–601.
