# Supplementary material for: A Low-Cost, Rapidly Integrated Debubbler (RID) Module for Microfluidic Cell Culture Applications
Source: Micromachines (Basel). 2019 May 30;10(6):360. doi: 10.3390/mi10060360 (PMC6632054; doi:10.3390/mi10060360)
Supplement: Supplementary file 1 [file micromachines-10-00360-s001.zip › MATLAB-script.zip!/RID_MATLAB_script_instructions.pdf]

A low-cost, rapidly integrated debubbler (RID) module for microfluidic cell culture applications

Matthew J. Williams[1], Nicholas K. Lee[1], Joseph A. Mylott[1], Nicole Mazzola[1], Adeel Ahmed[2] and Vinay V. Abhyankar[1]*
[1]Department of Biomedical Engineering, [2]Microsystems Engineering
Rochester Institute of Technology, Rochester NY 14623
*Correspondence: vvabme@rit.edu

Overview of MATLAB imaging script

The main script of analysis is the bubbleTrap function. The other scripts are subfunctions used by the main script. The analysis work flow is as follows:

1. bubbleTrap function receives video file, start time, and duration after start to be analyzed and sets up output vectors
2. Video passed to importVideo function which rotates, crops, grayscales each frame of the video, and generates time vector.
3. Individual frames are then passed to the filterImage, markImage, and measureImage functions
   a. Image filter is based on black white threshold value to binarize the image followed by an area min/max filter
   b. Image marker records the pixel column indices of the bubble edges.
   c. Image measure function finds the distances between bubble edges in pixels and converts to millimeters. Average bubble volume in microliters approximated using tubing inner diameter. Air to total volume ratio in percent also calculated.
4. The First mark of each frame passed to calculateFlowrate function which finds the average flow rate in milliliters per minute.
5. Final outputs are basic statistics about the bubble volumes, average air density, and flow rate.

Tutorial for video analysis:

1. Download all the MATLAB files into appropriate folder
   a. ***bubbleTrap.m***
   b. ***importVideo.m***
   c. ***filterImage.m***
   d. ***markImage.m***
   e. ***measureImage.m***
   f. ***calculateFlowrate.m***
2. Set parameters in ***importVideo*** script
   a. Place edited video into prefered MATLAB folder for use

b. Import video into workspace
c. Manually find correct cropping frame in MATLAB so that only the tubing with the bubbles is visible.(cropped to 75mm length of tubing in our analysis)
    i. Open up first frame of video using readFrame() and imshow() to use as a test frame

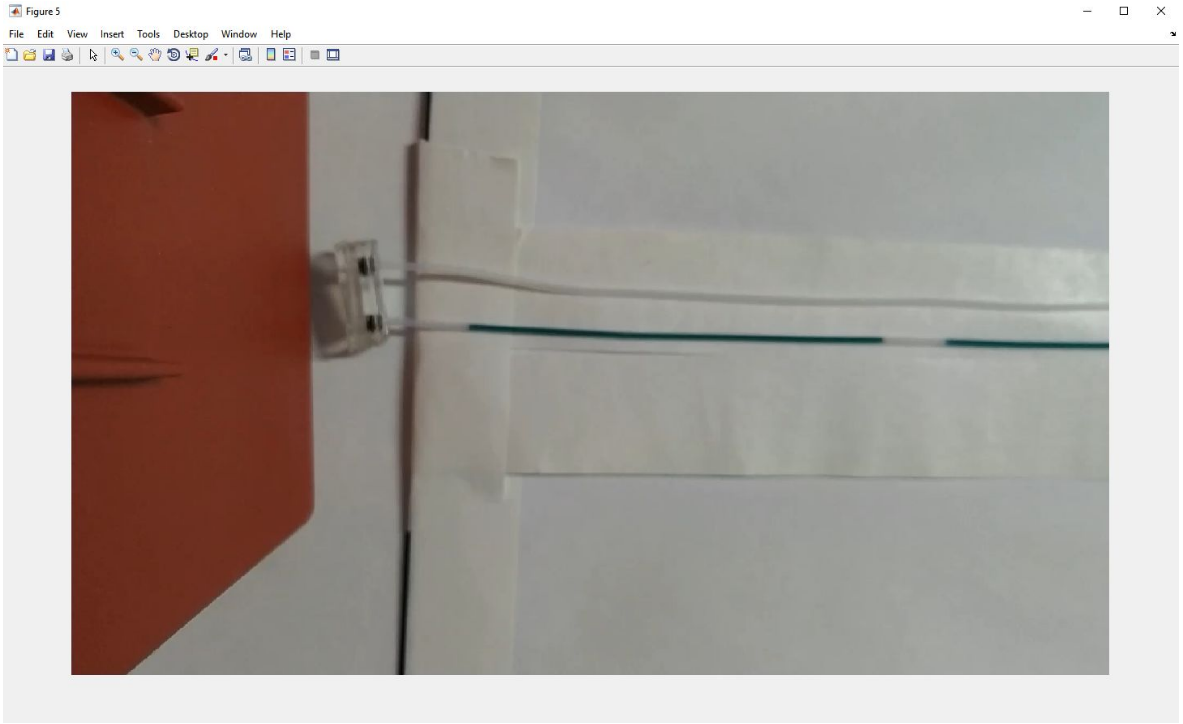

d. Rotate and edit the test frame of the video to fix the orientation and position of the tubing so that the tubing is horizontal.

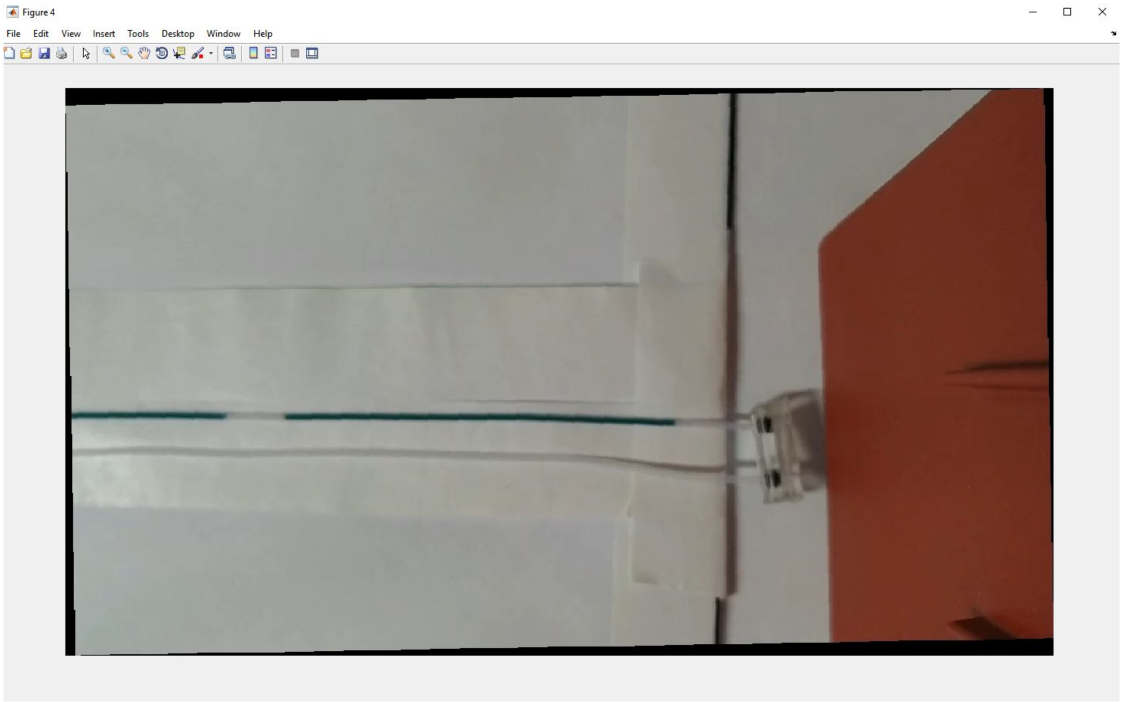

e. Use the data cursor to estimate the pixel position of the upper left corner desired tubing location
f. Use imcrop() to select only the tubing with the bubbles
g. Once the correct frame is determined, in the *importVideo* script, edit the **Cframe** vector in the parameter section to the appropriate values.

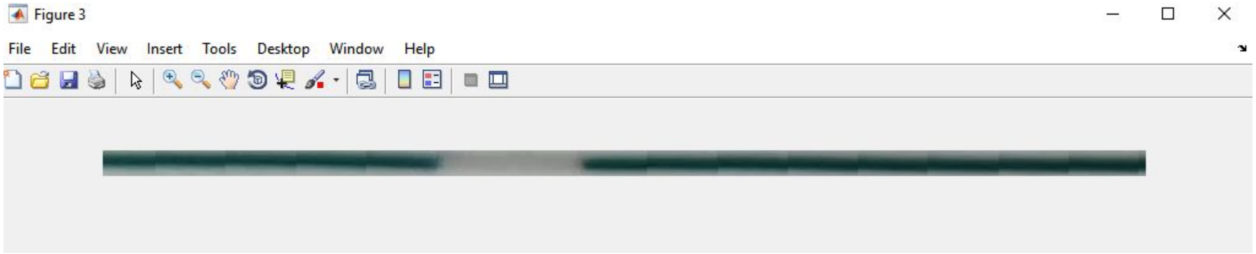

h. Change the value of the **spf** constant to match the seconds per frame of the video.

3. Set parameters in the ***bubbleTrap*** script
   a. Recolor test frame to grayscale with rgb2gray()
   b. Use the default parameters already in the *bubbleTrap* script to run the ***filterImage*** function on the test frame using command window.

      Ex. filterImage(data, bwthresh, areaFilt_min, areaFilt_max)
   c. Adjust parameters as needed until bubbles are clearly identified like the image below. Then edit **bwthresh, areaFilt_min,** and **areaFilt_max** as needed.

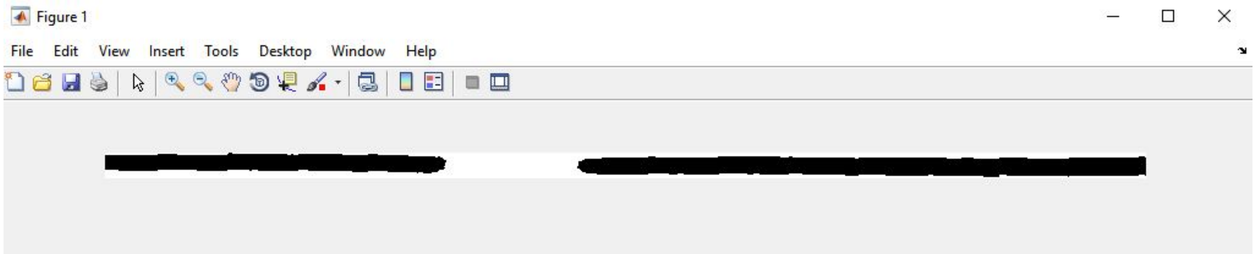

   d. Change the value of bubbleH_thresh to 1-5 pixels than the total height of the cropped image
      Ex. if total height = 20, bubbleH_thresh should be ~18
   e. Using the annotation function, mark a line on the very edge left edge of the test frame
   a. Then in ***bubbleTrap*** script, replace value of **imgStart** with the horizontal index value used in the annotation function
      Ex. annotation('line',[.084 .084],[0 1]);
            imgStart = 0.084;
4. Set parameters for measureImage script ***IMPORTANT***
   a. Enter in the mm to pixel ratio of the video as the value for **mm_pixel.** It is currently set assuming that the total width of the cropped image is 75mm but any other width or other ratio can be entered.
   b. Enter in the inner radius of the tubing used in the video in millimeters as the value for **tube_r**.
5. Set parameters for ***calculateFlowrate*** script ***IMPORTANT***
   a. Enter in the mm to pixel ratio.
   b. Enter in the inner radius of the tubing
6. Run ***bubbleTrap*** function in command window
      Ex. [sizeAvg,densityAvg,flow]=bubbleTrap('filename', start time, duration)
7. Video import function will take 30-90 seconds depending on how long the video is.
8. If the bubble verification section in the ***bubbleTrap*** function is not commented out, a figure will display each figure with the bubble edges marked with red lines. This section can be commented out to reduce analysis time, but should be used at least once to verify proper bubble identification.
